# Sympathetic activation by lower body negative pressure decreases kidney perfusion without inducing hypoxia in healthy humans

**DOI:** 10.1007/s10286-018-0570-7

**Published:** 2018-11-02

**Authors:** René van der Bel, Jasper Verbree, Oliver J. Gurney-Champion, Matthias J. P. van Osch, Erik S. G. Stroes, Aart J. Nederveen, C. T. Paul Krediet

**Affiliations:** 1 Department of Internal Medicine, Academic Medical Center at the University of Amsterdam, Meibergdreef 9, 1105AZ Amsterdam, The Netherlands; 2 Department of Radiology, C.J. Gorter Center for High Field MRI, Leiden University Medical Center, Leiden, The Netherlands; 3 Department of Radiology, Academic Medical Center at the University of Amsterdam, Amsterdam, The Netherlands; 4 Department of Radiation Oncology, Academic Medical Center at the University of Amsterdam, Amsterdam, The Netherlands

**Keywords:** Lower body negative pressure, Sympathetic nervous system, Kidney oxygenation, BOLD MRI, Kidney perfusion, Blood pressure

## Abstract

**Purpose:**

There is ample evidence that systemic sympathetic neural activity contributes to the progression of chronic kidney disease, possibly by limiting renal blood flow and thereby inducing renal hypoxia. Up to now there have been no direct observations of this mechanism in humans. We studied the effects of systemic sympathetic activation elicited by a lower body negative pressure (LBNP) on renal blood flow (RBF) and renal oxygenation in healthy humans.

**Methods:**

Eight healthy volunteers (age 19–31 years) were subjected to progressive LBNP at − 15 and − 30 mmHg, 15 min per level. Brachial artery blood pressure was monitored intermittently. RBF was measured by phase-contrast MRI in the proximal renal artery. Renal vascular resistance was calculated as the MAP divided by the RBF. Renal oxygenation (R2*) was measured for the cortex and medulla by blood oxygen level dependent (BOLD) MRI, using a monoexponential fit.

**Results:**

With a LBNP of − 30 mmHg, pulse pressure decreased from 50 ± 10 to 43 ± 7 mmHg; MAP did not change. RBF decreased from 1152 ± 80 to 1038 ± 83 mL/min to 950 ± 67 mL/min at − 30 mmHg LBNP (*p* = 0.013). Heart rate and renal vascular resistance increased by 38 ± 15% and 23 ± 8% (*p* = 0.04) at − 30 mmHg LBNP, respectively. There was no change in cortical or medullary R2* (20.3 ± 1.2 s^−1^ vs 19.8 ± 0.43 s^−1^; 28.6 ± 1.1 s^−1^ vs 28.0 ± 1.3 s^−1^).

**Conclusion:**

The results suggest that an increase in sympathetic vasoconstrictor drive decreases kidney perfusion without a parallel reduction in oxygenation in healthy humans. This in turn indicates that sympathetic activation suppresses renal oxygen demand and supply equally, thus allowing adequate tissue oxygenation to be maintained.

## Introduction

Systemic hyperactivity of the sympathetic nervous system is a hallmark of chronic kidney disease (CKD) [7, 9, 15]. Moreover, sympathetic nerve activity (SNA) is an independent predictor of kidney disease progression [18]. Also, therapies that limit sympathetic nervous system activity have shown to improve kidney function [7]. This has led to the pathophysiological paradigm that sympathetic activity is a causal factor in the progression of CKD [15, 18, 20, 27, 30]. Central to the mechanism by which this occurs are the direct stimulation of profibrotic factors [13] and the induction of hypoxia [16, 30], which is the topic of the current study.

The renal parenchyma is characterized by a steep pO_2_ gradient and is thereby susceptible to hypoxia [5, 21, 22]. In animals, renal sympathetic activation decreases renal blood flow [14]. Simultaneously, sympathetic nerves directly innervate the renal tubules, inducing sodium reabsorption and thus increasing metabolic demand [3, 21]. The net effect of decreased renal blood flow and increased tubular demand is therefore a decrease in oxygenation [3, 6, 21]. However, direct observations of the effect of sympathetic activation on renal oxygenation in humans are lacking.

Lower body negative pressure (LBNP) can be used to experimentally increase systemic SNA in humans [2, 10, 12]. Low-grade LBNP induces sustained sympathetic activation without systemic blood pressure effects [12]. Moderate-grade LBNP induces further sympathetic activation with moderate hemodynamic effects while maintaining organ perfusion pressure [10, 26, 28]. In the kidneys, LBNP reduces blood flow and the glomerular filtration rate while the glomerular filtration fraction (FF) remains unaffected [8, 28, 33]. LBNP is therefore ideally suited to investigate sympathorenal effects on renal oxygenation.

Kidney oxygenation can reliably be assessed by blood oxygen level dependent (BOLD) MRI [24, 25]. As BOLD MRI is sensitive to the blood deoxyhemoglobin level, the acquired signal is the composite result of oxygen extraction from the blood (i.e., metabolic demand) and the rate of oxygen delivery (i.e., perfusion) [17]. This technique was originally validated in a porcine model [23]. The technique was also shown to provide excellent intra-individual tracking of minor changes in kidney oxygenation in subsequent human studies. For example, we showed a 5% decrease in cortical oxygenation during angiotensin II (Ang-II) infusion in healthy humans. These changes are caused by an Ang-II-driven increase in FF, i.e., increasing tubular workload relative to renal perfusion [29].

There seems to be a conflict between two (patho)physiologic observations regarding the role of SNA in the kidney hypoxia. On the one hand, sympathetic activation decreases renal blood flow and increases tubular metabolic load, affecting renal oxygenation negatively [6]. On the other hand, sympathetic activity does not alter FF [8, 28, 33], thereby maintaining the balance between metabolic demand and oxygen supply. Against this background, we set out to explore the physiological effect of sympathetic activation by LBNP on cortical and medullary oxygenation by BOLD MRI in healthy human subjects. We hypothesized that with low to moderate grades of LBNP, renal blood flow and medullary oxygenation decreases, while cortical oxygenation is only affected at moderate-grade LBNP. In addition, we compared the renal oxygenation effects of LBNP to those induced by Ang-II using historic data.

## Materials and methods

### Study population

Eight healthy subjects were studied (age 19–31 years, 5/3 male/female, height 1.61–1.85 m, weight 62–76 kg). Their medical histories revealed no significant disease and none used medication. Written informed consent was obtained from all participants, and the study protocol (protocol number NL53367.018.15) was approved by the Medical Ethics Review Committee of the Academic Medical Center (METC AMC, Amsterdam, The Netherlands). The study was conducted in accordance with the Declaration of Helsinki 2013.

### Study design

**Fig. 1:**
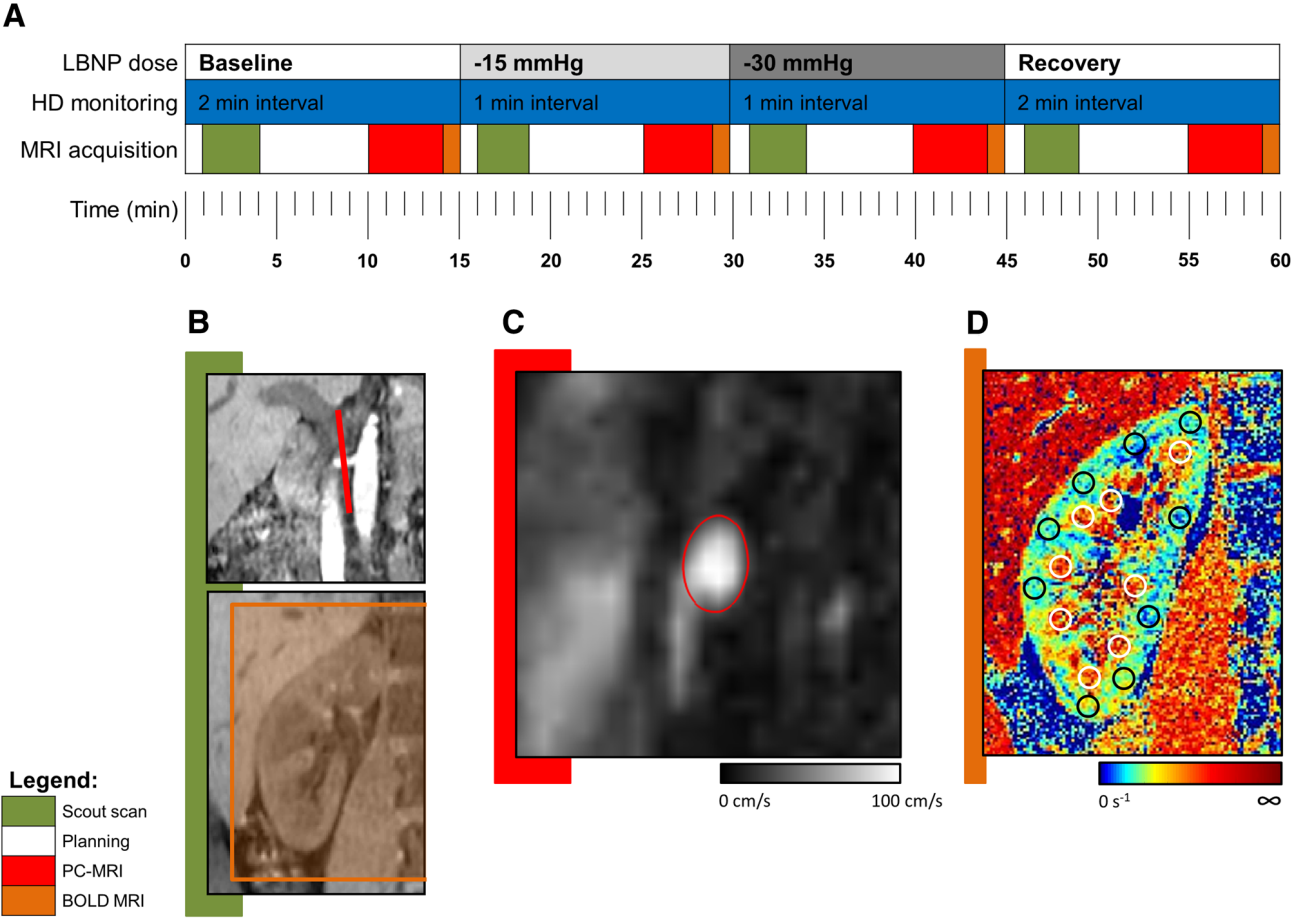
Overview of the study design. **a** Timeline of the LBNP experiments, with the sequence of MRI acquisitions (*green* scout scan,* red* phase-contrast MRI,* orange* BOLD MRI). **b–d** Examples of the images acquired: scout scans with PC and BOLD MRI planning (**b**), velocity-encoded (*V*_enc_) map with segmented proximal renal artery (**c**), and R2* map of coronal BOLD MRI slice with cortical and medullary segmentations (**d**)

Kidney MRI was performed at baseline and during consecutive LBNP levels at − 15, − 30 mmHg, and recovery to 0 mmHg for 15 min each. The timeline of the protocol is given in Fig. 1. Based on data from previous studies [8, 28, 33], the LBNP protocol was designed to reduce renal perfusion by LBNP to a similar extent to that achieved by continuous Ang-II infusion (0.3, 0.9 ng/kg/min) in our previous study of a different group of eight similarly healthy subjects (age 19–22 years, 5/3 male/female, height 1.61–1.90 m, weight 63–82 kg) with identical MRI acquisitions [29]. This enabled comparison between the oxygenation responses of sympathetic activity (by LBNP) to Ang-II.

After being instrumented with ECG electrodes and a brachial artery cuff, the subjects were placed in a custom-built LBNP box (Department of Medical Technology, LUMC, The Netherlands) mounted on the MRI table. The box had a fixed saddle and was sealed around the subjects’ waists using a neoprene seal just above the iliac crest. Pillows, towels, and sandbags were used to provide a fully comfortable body position. An anterior MRI coil was secured over chest and abdomen. During the LBNP challenge, blood pressure and heart rate were monitored intermittently at ≥ 2-min intervals (Accutor Plus, Datascope Corp., USA). For subject safety, there was direct two-way audio communication with the LBNP vacuum pump and MRI operators. Also, a physician was present inside the MRI room to directly observe the subjects for signs of discomfort and/or presyncope.

### Magnetic resonance imaging

Magnetic resonance imaging was performed on a 3.0-T MRI system (Ingenia, Philips Healthcare, Best, Netherlands), as described previously [29]. Survey scans, including 3D T1-weighted multi-echo gradient echo (T1 W GE) with Dixon reconstruction [4], were used to locate the positions of the kidneys and renal arteries. To account for LBNP-associated motion of the subject, the Dixon-reconstructed survey scan was repeated at every LBNP dose. BOLD and phase-contrast (PC) MRI scans were subsequently acquired at baseline and during each LBNP dose.

Three-directional blood flow velocity was measured by PC MRI in a slice placed perpendicular to the right proximal renal artery, as described previously [1, 29]. In short, the PC MRI sequence parameters were as follows: the number of ECG-triggered cardiac phases was 30, field of view (FOV) = 200 × 200 mm, resolution = 0.65 × 0.65 mm^2^, slice thickness = 3 mm, repetition time (TR) /echo time (TE) = 8.5/5.7 ms, flip angle = 10°, SENSE factor = 2 (right-left direction), *V*_enc_ = 100 cm/s in all directions. Acquisition time was 3:45 min, and the sequence was acquired during free breathing. Offline image processing for PC MRI was performed using dedicated software (GTF low version 2.2.9, Gyro Tools LLC, Switzerland). After correcting for background phase-offset errors and aliasing, the mean RBF (mL/s) was calculated using manual vessel segmentation in each cardiac phase. For further RBF analysis, equal perfusion of both the right and the left kidneys was assumed [11].

Changes in the BOLD MRI signal were quantitatively assessed by measuring the transverse relaxation rate (R2*) within different regions of interest (ROIs). We measured R2* using a multi-echo single-slice gradient-echo MRI sequence with the following parameters: FOV = 400 × 400 mm^2^, resolution = 1.2 × 1.2 mm^2^, slice thickness = 4 mm, TR = 140 ms, flip angle = 70°, TE_1_ = 2 ms, ΔTE = 5 ms; number of echoes = 16 [25]. Image acquisition was performed in 18 s during a single expiratory breath hold in a coronal slice where the kidney cross-section was largest and cortical/medullary definition was best on the survey scans. For BOLD MRI analysis, circular ROIs with a diameter of eight voxels were defined at regular intervals throughout each kidney’s cortex and medulla in the baseline scan. For each subject, the resulting masks were then transferred to the three subsequent BOLD images. Renal R2* values were calculated for cortex and medulla separately, using monoexponential fits [25, 29] obtained with routines written in Matlab (The MathWorks, Natick, USA).

### Study power and statistical analysis

The study was powered based on previous data using the same MRI protocols in healthy humans [29]. In that study, we found that a 20% perfusion reduction resulted in a 2.4 s^−1^ increase in cortical R2*. Anticipating a potentially reduced effect or potentially more variance in the current experiment, this study was powered at 0.81 to detect an R2* increase of 1.4 s^−1^ in *n* = 8.

All data are presented as the mean with the standard error. Renal vascular resistance (RVR) was calculated as the mean arterial pressure (MAP) divided by the renal blood flow (RBF). The Shapiro–Wilk test was used to verify that the data were distributed normally. One-way ANOVA for repeated measures was used to assess the dose effect of LBNP on systemic hemodynamic effects (blood pressure, heart rate) and renal (RVR, RBF) hemodynamic parameters as well as cortical and medullary R2*. A *Z*-test was used to compare the LBNP response to the historic positive control (Ang-II response). The *z*-score is reported with a two-tailed *p* value. All statistical analyses were performed using SPSS Statistics 22 (IBM, Chicago, USA). A significance level of *α* = 0.05 was used.

## Results

### Subjects

All subjects tolerated the LBNP doses well and reported no periods of discomfort or lightheadedness. Baseline measurements are given in Table 1.

**Table 1:** Baseline characteristics per subject

Subject (#, ♂/♀)	Hemodynamics			MRI		
	MAP (mmHg)	HR (bpm)	RVR (mL/min/mmHg)	CR2* (s^−^^1^)	MR2* (s^−^^1^)	RBF (mL/min)
1, ♂	79	66	0.064	20.2	33.0	1229
2, ♂	85	82	0.059	17.0	24.9	1430
3, ♀	81	65	0.065	18.6	29.5	1242
4, ♀	83	67	0.082	24.0	25.9	1015
5, ♂	81	77	0.093	21.4	27.5	869
6, ♂	86	64	0.060	17.6	29.9	1438
7, ♀	98	86	0.11	17.9	25.8	856
8, ♂	87	61	0.077	26.2	32.0	1133
Mean (SEM)	85 (2.1)	71 (3.3)	0.076 (0.0063)	20.3 (1.1)	28.6 (1.0)	1152 (75)

Baseline characteristics after 15 min of supine rest

*bpm* beats per minute, CR2* cortical R2*, *HR* heart rate, *MAP* mean arterial pressure, MR2* medullary R2*, *MRI* magnetic resonance imaging, *RBF* renal blood flow, *RVR* renal vascular resistance

### Systemic hemodynamic changes induced by LBNP

**Fig. 2:**
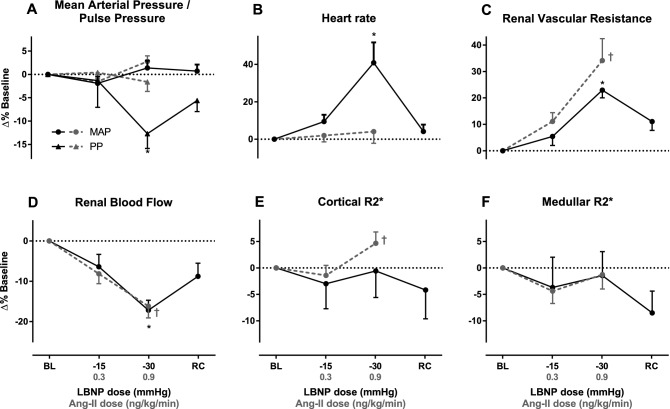
Systemic and renal hemodynamic effects of low- to moderate-grade LBNP.** a** Mean arterial pressure/pulse pressure.** b** Heart rate.** c** Renal vascular resistance.** d** Renal blood flow.** e** Cortical R2*.** f** Medullar R2*. All graphs depict the percent change compared to baseline (BL) at − 15mmHg LBNP, − 30 mmHg LBNP, and during recovery (*RC*). Graphs depict the results of the current study in* black* and the results of our previous study using Ang-II infusion in* gray*, for comparison. That study assessed the same parameters as a function of the continuous infusion of Ang-II. Significant responses as assessed by repeated-measures ANOVA are indicated by * and † for LBNP and Ang-II infusion, respectively [28]

The absolute values of the hemodynamic parameters during each stage of the experiment are listed in Table 2. Figure 2 depicts the responses in percentage change compared to baseline. As expected, the arterial blood pressure did not change significantly at the chosen levels of LBNP. Only the pulse pressure decreased significantly, from 50 ± 3.5 at baseline to 43 ± 2.3 mmHg at − 30 mmHg, *F*_(2,14)_ = 6.4, *p* = 0.043 (Fig. 2a). The heart rate increased by 38% ± 15% at maximum LBNP (*F*_(2,14)_ = 20.1, *p* = 0.004, Fig. 2b). These hemodynamic effects were present in all individual subjects. The LBNP intervention did not induce (pre)syncope in any of the subjects.

**Table 2:** Effects of LBNP

	Hemodynamics						MRI		
	SBP (mmHg)	DBP (mmHg)	MAP (mmHg)	PP (mmHg)	HR (bpm)	RVR (mL/min/mmHg)	CR2* (s^−^^1^)	MR2* (s^−^^1^)	RBF (mL/min)
Baseline	118 (3.3)	69 (2.1)	85 (2.0)	50 (3.5)	71 (3.1)	0.076 (0.0063)	20.3 (1.1)	28.6 (1.0)	1152 (75)
LBNP − 15 mmHg	114 (2.8)	68 (2.3)	83 (2.1)	47 (2.7)	76 (2.6)	0.083 (0.0057)	19.6 (0.84)	28.0 (1.8)	1039 (72)
LBNP − 30 mmHg	115 (2.6)	72 (1.9)	86 (1.9)	43 (2.3)*	90 (2.6)*	0.094 (0.0067)*	19.8 (0.40)	28.0 (1.2)	950 (63)*
Recovery	116 (2.9)	70 (2.0)	85 (2.0)	45 (2.6)	72 (2.3)	0.088 (0.0073)	19.6 (0.56)	26.5 (0.88)	1012 (68)

*bpm* beats per minute, CR2* cortical R2*, *HR* heart rate, *MAP* mean arterial pressure, MR2* medullary R2*, *MRI* magnetic resonance imaging, *PP* pulse pressure, *RBF* renal blood flow, *RVR* renal vascular resistance

*Indicates significant response to LBNP

### Renal perfusion and oxygenation

The absolute values of MRI-derived perfusion and oxygenation parameters during each stage of the experiment are listed in Table 2. Figure 2 depicts the responses in percentage change compared to baseline. Renal vascular resistance increased as a function of LBNP level by 23 ± 8% at − 30 mmHg LBNP (*F*_(2,14) _= 27.8, *p* = 0.002, Fig. 2c). RBF decreased by 17% ± 2% at − 30 mmHg LBNP, *F*_(2,14) _= 11.8, *p* = 0.013, Fig. 2d, black line). These renal hemodynamic effects were observed in all individual subjects. Neither the cortical R2* (CR2*) nor the medullary R2* (MR2*) changed during LBNP (Fig. 2e, f, black lines).

### LBNP compared to Ang-II response

In a previous study, we performed an experiment similar to the current study using Ang-II infusion. The data from that experiment are depicted in gray in Fig. 2 to aid comparison of the different effects of these interventions. In the Ang-II study, we measured perfusion and R2* changes induced by 0.3 and 0.9 ng/kg/min Ang-II infusion in healthy volunteers [29]. In those experiments, RBF showed an Ang-II dose-dependent decrease from 1215 ± 83 to 1025 ± 89 mL/min (Fig. 2d, gray line) and cortical R2* increased from 17.4 ± 1.1 to 19.3 ± 0.8 s^−1^ (Fig. 2e, gray line). The average cortical R2* response was *β* = 0.025 ± 0.17 in the LBNP group and *β* = 5.9 ± 2.4 in the Ang-II group; these responses were significantly different, with a *z*-score of *z* = 2.5 and a two-tailed *p* value of *p* = 0.014.

## Discussion

Our findings can be summarized as follows. LBNP consistently increased heart rate and lowered pulse pressure, while organ perfusion pressure (MAP) was unchanged in all subjects. This is consistent with selective sympathetic activation. LBNP induced a marked increase in renal vascular resistance and reduced renal perfusion substantially in all subjects. However, during selective sympathetic activation, there was no discernible effect on kidney oxygenation in the cortex or medulla. This is in contrast to the decreased cortical oxygenation upon Ang-II infusion, with similar changes in renal vascular resistance and flow. These explorative data do not support sympathetic activity as a causal factor of renal hypoxia under physiological circumstances.

In light of the two conflicting concepts regarding the possible influences of SNA on renal oxygenation (decreased RBF with increased metabolic load vs. a maintained FF and oxygenation balance), we observe the following. During our LBNP experiments, we found a renal vascular resistance increase and a perfusion reduction that were virtually identical to those found by other authors in previous studies involving healthy humans [8, 28, 32, 33]. In those studies, both ERPF and GFR (as assessed by radioisotope measurements) decreased proportionally by approximately 10 and 20% at − 15 and − 30 mmHg, respectively, while FF was maintained. Applying those observations to our study, we infer that the oxygenation balance was maintained, which explains the absence of an effect of LBNP-induced sympathetic activation on renal oxygenation. This is further supported by Würzner et al., who also reported that the fractional distal reabsorption of sodium was unaffected during LBNP [33]. Distal sodium reabsorption in the medullary thick ascending limb of Henle’s loop is metabolically demanding [21], and it seems that this process is also affected by LBNP in proportion to the renal perfusion reduction [33]. Thus, the two processes with the most influence on renal oxygenation status, i.e., filtration fraction and distal sodium reabsorption, are affected in the same direction and to an equal extent to the RBF reduction during LBNP. This would suggest that sympathetic activation per se does not influence renal oxygenation in healthy humans.

Despite the known intricate interplay between the sympathetic nervous system and the renin angiotensin system (RAS), the current observations stand in contrast to the effects of Ang-II on renal oxygenation. Contrary to direct sympathetic neural activation, Ang-II directly changes the balance between oxygen supply and demand through vasoconstriction of the efferent arterioles of the glomerulus. Ang-II thereby decreases renal perfusion while metabolic demand is maintained [29].

This is the first study to directly measure renal oxygenation during sympathetic activation in healthy humans. We successfully used a LBNP intervention as a sympathetic stimulus, in combination with renal MRI measurements. Although the imaging area was in close proximity to the LBNP box, we did not observe any distortion in the MR images. The premise of this study was a universal physiological phenomenon. The lack of an effect on cortical and medullary R2* may suggest a potential type II statistical error. We acknowledge that the small sample size is the most important limitation of our study. However, a post hoc power calculation for the point estimate from the current study showed that number of subjects needed to achieve a statistical power of 80% to consolidate this effect is 209. Apart from the questionable relevance of such a minor effect, it is not feasible to include this number of subjects. We speculate that, in CKD patients, the effects may be discernable in smaller sample sizes.

A limitation related to the small sample size is the lack of a comparison between males and females. Although each subject is its own control, effects of male/female differences may be reflected in the variation within the results. Other limitations of our study concern the absence of direct verification of an increased sympathetic tone (e.g., by microneurography) and the degree of sodium retention during the experiments. However, these effects have been extensively documented [8, 10, 12, 26, 28, 33], and combining these measurements with MRI is not (yet) feasible. Another limitation is that we cannot rule out that other factors were superimposed on the sympathetic neural activation. Specifically, we did not measure hormones that regulate renal hemodynamics, while it is known that RAS activation and renal noradrenaline spillover occur at medium-grade LBNP. It has been documented that 10 min of a LBNP of − 18 mmHg or lower induces detectable increases in plasma renin activity and Ang-II levels [28]. Renal noradrenaline spillover has been shown to increase in conjunction with sympathetic nerve activity [19]. Possibly, RAS activation and/or noradrenaline spillover is reflected in the delayed return to baseline in renal perfusion and renal vascular resistance after LBNP cessation that we observed. In these experiments, the RAS activation kinetics induced by LBNP may have been different or insufficient to induce renal hypoxia. Another possible underlying effect of LBNP that may be involved in the described mechanisms is the difference in response between cardiac and renal sympathetic outflow. It has been shown in sheep that organ-specific responses are regulated through different neural pathways [31]. Our results cannot discern between those specific neural effects. In general, MR imaging may affect sympathetic tone due to the confined surroundings, movement restriction, loud noises, and end-expiratory breath holds required for BOLD-MRI. However, these circumstances were constant during all stages of the experiments and their effects on individual outcomes were expected to be minimal, and none of the subjects suffered from claustrophobia. Lastly, the data from a previous experiment presented in the results and discussed above were obtained from a different group of subjects who had been recruited from the same population as the subjects in the current study.

Regarding the generalizability of our results to CKD patients, we are limited by the fact that this constitutes an acute experiment in a small group of young healthy subjects versus a chronic pathophysiological process observed in patients. However, a chronically sustained sympathetic activating intervention is not feasible in humans. Whether the renal oxygenation response to LBNP is different in patients should be explored in further studies.

In conclusion, our exploratory data question the universal physiological concept that sympathetic hyperactivity per se decreases kidney oxygenation. We showed that selective induction of sympathetic activity by LBNP induces a substantial and consistent renal blood flow reduction without parallel cortical or medullary hypoxia. These data are in agreement with the notion that sympathetic activation suppresses renal oxygen demand and supply equally, thus maintaining adequate tissue oxygenation.

## References

[1] Bax L, Bakker CJ, Klein WM, Blanken N, Beutler JJ, Mali WP (2005). Renal blood flow measurements with use of phase-contrast magnetic resonance imaging: normal values and reproducibility. J Vasc Interv Radiol.

[2] Davy KP, Seals DR, Tanaka H (1998). Augmented cardiopulmonary and integrative sympathetic baroreflexes but attenuated peripheral vasoconstriction with age. Hypertension.

[3] DiBona GF, Kopp UC (1997). Neural control of renal function. Physiol Rev.

[4] Dixon WT (1984). Simple proton spectroscopic imaging. Radiology.

[5] Evans RG, Goddard D, Eppel GA, O’Connor PM (2011). Factors that render the kidney susceptible to tissue hypoxia in hypoxemia. Am J Physiol Regul Integr Comp Physiol.

[6] Evans RG, Ince C, Joles JA, Smith DW, May CN, O’Connor PM, Gardiner BS (2013). Haemodynamic influences on kidney oxygenation: clinical implications of integrative physiology. Clin Exp Pharmacol Physiol.

[7] Ewen S, Ukena C, Linz D, Schmieder RE, Bohm M, Mahfoud F (2013). The sympathetic nervous system in chronic kidney disease. Curr Hypertens Rep.

[8] Gilbert CA, Bricker LA, Springfield WT, Stevens PM, Warren BH (1966). Sodium and water excretion and renal hemodynamics during lower body negative pressure. J Appl Physiol.

[9] Grassi G, Bertoli S, Seravalle G (2012). Sympathetic nervous system: role in hypertension and in chronic kidney disease. Curr Opin Nephrol Hypertens.

[10] Hirsch AT, Levenson DJ, Cutler SS, Dzau VJ, Creager MA (1989). Regional vascular responses to prolonged lower body negative pressure in normal subjects. Am J Physiol.

[11] Hura C, Stein JH (2011) Renal blood flow. In: Comprehensive physiology. Wiley, Hoboken, pp 1129–1118

[12] Joyner MJ, Shepherd JT, Seals DR (1990). Sustained increases in sympathetic outflow during prolonged lower body negative pressure in humans. J Appl Physiol.

[13] Kim J, Padanilam BJ (2013). Renal nerves drive interstitial fibrogenesis in obstructive nephropathy. J Am Soc Nephrol.

[14] Koeners MP, Vink EE, Kuijper A, Gadellaa N, Rosenberger C, Mathia S, van den Meiracker AH, Garrelds IM, Blankestijn PJ, Joles JA (2014). Stabilization of hypoxia inducible factor-1alpha ameliorates acute renal neurogenic hypertension. J Hypertens.

[15] Koomans HA, Blankestijn PJ, Joles JA (2004). Sympathetic hyperactivity in chronic renal failure: a wake-up call. J Am Soc Nephrol.

[16] Leonard BL, Malpas SC, Denton KM, Madden AC, Evans RG (2001). Differential control of intrarenal blood flow during reflex increases in sympathetic nerve activity. Am J Physiol Regul Integr Comp Physiol.

[17] Liss P, Cox EF, Eckerbom P, Francis ST (2013). Imaging of intrarenal haemodynamics and oxygen metabolism. Clin Exp Pharmacol Physiol.

[18] Masuo K, Lambert GW, Esler MD, Rakugi H, Ogihara T, Schlaich MP (2010). The role of sympathetic nervous activity in renal injury and end-stage renal disease. Hypertens Res.

[19] May CN, Frithiof R, Hood SG, McAllen RM, McKinley MJ, Ramchandra R (2010). Specific control of sympathetic nerve activity to the mammalian heart and kidney. Exp Physiol.

[20] Neumann J, Ligtenberg G, Klein II, Koomans HA, Blankestijn PJ (2004). Sympathetic hyperactivity in chronic kidney disease: pathogenesis, clinical relevance, and treatment. Kidney Int.

[21] O’Connor PM (2006). Renal oxygen delivery: matching delivery to metabolic demand. Clin Exp Pharmacol Physiol.

[22] Pallone TL, Silldorff EP, Turner MR (1998). Intrarenal blood flow: microvascular anatomy and the regulation of medullary perfusion. Clin Exp Pharmacol Physiol.

[23] Pedersen M, Dissing TH, Morkenborg J, Stodkilde-Jorgensen H, Hansen LH, Pedersen LB, Grenier N, Frokiaer J (2005). Validation of quantitative BOLD MRI measurements in kidney: application to unilateral ureteral obstruction. Kidney Int.

[24] Pruijm M, Hofmann L, Maillard M, Tremblay S, Glatz N, Wuerzner G, Burnier M, Vogt B (2010). Effect of sodium loading/depletion on renal oxygenation in young normotensive and hypertensive men. Hypertension.

[25] Rossi C, Sharma P, Pazahr S, Alkadhi H, Nanz D, Boss A (2013). Blood oxygen level-dependent magnetic resonance imaging of the kidneys: influence of spatial resolution on the apparent R2* transverse relaxation rate of renal tissue. Invest Radiol.

[26] Rowell LB, Seals DR (1990). Sympathetic activity during graded central hypovolemia in hypoxemic humans. Am J Physiol.

[27] Siddiqi L, Joles JA, Grassi G, Blankestijn PJ (2009). Is kidney ischemia the central mechanism in parallel activation of the renin and sympathetic system?. J Hypertens.

[28] Tidgren B, Hjemdahl P, Theodorsson E, Nussberger J (1990). Renal responses to lower body negative pressure in humans. Am J Physiol.

[29] van der Bel R, Coolen BF, Nederveen AJ, Potters WV, Verberne HJ, Vogt L, Stroes ES, Krediet CT (2016) Magnetic resonance imaging-derived renal oxygenation and perfusion during continuous, steady-state angiotensin-II infusion in healthy humans. J Am Heart Assoc 5:e003185 10.1161/JAHA.115.003185 PMC4943284 27021686

[30] Vink EE, Blankestijn PJ (2012). Evidence and consequences of the central role of the kidneys in the pathophysiology of sympathetic hyperactivity. Front Physiol.

[31] Wallin BG, Thompson JM, Jennings GL, Esler MD (1996). Renal noradrenaline spillover correlates with muscle sympathetic activity in humans. J Physiol (Lond).

[32] Wuerzner G, Chiolero A, Maillard M, Nussberger J, Burnier M (2005). Metoprolol prevents sodium retention induced by lower body negative pressure in healthy men. Kidney Int.

[33] Wurzner G, Chiolero A, Maillard M, Nussberger J, Hayoz D, Brunner HR, Burnier M (2001). Renal and neurohormonal responses to increasing levels of lower body negative pressure in men. Kidney Int.

